# Dr. Gibbes, on the Bath Waters

**Published:** 1806-11

**Authors:** G. S. Gibbes

**Affiliations:** 25, Circus, Bath


### *To the Editors of the Medical and Phyſical Journal.*

GENTLEMEN,

AS you have done me the honour of noticing some late experiments of mine on the Bath waters, and as you have omitted to mention some results, which appear to me of importance, I take the liberty of sending you the following account:

Having frequent opportunities of witnessing the powerful effects of the Bath waters when taken internally, and having often seen how analogous those effects were to those resulting from the use of steel medicines, I have been much perplexed to account for their action; since the ordinary tests of iron shew that they contain so small a portion of that mineral. The quantity of iron acted upon by the tincture of galls is so small, that we could not attribute with any degree of satisfaction their medicinal powers to it. According to Mr. Phillips, not more than one sixty-eighth part of a grain of oxide of iron is contained* in one pint of the water. I have frequently seen very strongly marked effects produced by so small a quantity of these waters as four ounces; and in those cases wherein the waters have been thus powerful, the symptoms produced by them have always been of the same kind; they have produced head-ache, giddiness, and a great de[illegible]rmination of blood to the head. Where they have thus [illegible]ed, I have compared their effects with ordinary water [illegible]he same temperature, and I have constantly found that it it was to the peculiar qualities of the Bath waters that we were to attribute these powerful symptoms. As the tincture of galls and the other tests of iron did not indicate any quantity of iron at all proportionate to such effects, I was led to suppose, that iron might exist either dissolved or suspended in them in a state not to be detected by those tests. With a view, therefore, to discover the nature of their ingredients, I very carefully examined all the substances that are deposited in and about the baths, channels, and reservoirs, and I found iron existing in three different states; on the glasses used at the pumps, on the sides and bottoms of the baths in state of yellow oxide, or carbonate; in the channels of the baths are found pyritical incrustations, which are vitriolizable on exposure to air and moisture; and, in the sand which accumulates in the reservoir under the bath, I found innumerable black particles of iron, which in the most lively manner obey the magnetic influence.

It appeared of importance to ascertain how far the waters held in solution iron that could thus produce these several appearances. I evaporated twenty-six gallons of water, and I procured 2252 grains of solid residuum. This residuum, when perfectly dry, I examined with a microscope, and I saw interspersed throughout the mass numberless black particles, and I found that on presenting a magnet to them they very readily and very decidedly were attracted and held up by it. I repeated the experiment on the same quantity of water, and I found that the slower the evaporation proceeded, the larger were those particles of iron which obeyed the magnetic influence. I must observe, that no iron vessel, or instrument containing iron, was used in this experiment.

I believe it is admitted, that the nearer iron approaches the metallic state, and is suspended or dissolved in water, the more decided are its medicinal effects, as in the green oxide of Griffith's mixture, &c.

Now the iron in the Bath waters being still nearer the metallic state, and being in that state dissolved in them, may be the reason why small quantities of these waters produce such powerful effects on some constitutions. The quantity of iron hitherto estimated as the quantity contained in these waters has been only that portion which is held in solution by the carbonic acid, and which is deposited on their cooling; and this is the reason why the Bath waters cease to give any signs of iron when tincture

 of of galls is added to them, after they have been cooled in contact with the external air.

I am, &c.

*25, Circus, Bath,*

*Oct.* 3, 1806.

G. S. GIBBES.

